# Differential Effects of Obesity, Hyperlipidaemia, Dietary Intake and Physical Inactivity on Type I versus Type IV Allergies

**DOI:** 10.3390/nu14112351

**Published:** 2022-06-05

**Authors:** Nathalie Rohmann, Liasita Munthe, Kristina Schlicht, Corinna Geisler, Tobias J. Demetrowitsch, Corinna Bang, Julia Jensen-Kroll, Kathrin Türk, Petra Bacher, Andre Franke, Karin Schwarz, Dominik M. Schulte, Matthias Laudes

**Affiliations:** 1 Institute of Diabetes and Clinical Metabolic Research, University Medical Center Schleswig-Holstein, Campus Kiel, 24105 Kiel, Germany; nathalie.rohmann@uksh.de (N.R.); liasitakarina@gmail.com (L.M.); kristina.schlicht@uksh.de (K.S.); corinna.geisler@uksh.de (C.G.); kathrin.tuerk@uksh.de (K.T.); dominik.schulte@uksh.de (D.M.S.); 2 Division of Food Technology, Department of Human Nutrition, Kiel University, 24105 Kiel, Germany; tdemetrowitsch@foodtech.uni-kiel.de (T.J.D.); jjensenkroll@foodtech.uni-kiel.de (J.J.-K.); kschwarz-2@foodtech.uni-kiel.de (K.S.); 3 Institute of Clinical Molecular Biology, Kiel University, 24105 Kiel, Germany; c.bang@ikmb.uni-kiel.de (C.B.); petra.bacher@uksh.de (P.B.); a.franke@ikmb.uni-kiel.de (A.F.); 4 Institute of Immunology, University Medical Center Schleswig-Holstein, Campus Kiel, 24105 Kiel, Germany; 5 Division of Endocrinology, Diabetes and Clinical Nutrition, Department of Internal Medicine I, University Medical Center Schleswig-Holstein, Campus Kiel, 24105 Kiel, Germany

**Keywords:** allergy, obesity, hyperlipidaemia, diet and lifestyle

## Abstract

Background: Alongside metabolic diseases (esp. obesity), allergic disorders are becoming increasingly prevalent. Since both obesity and allergies are highly impacted by environmental determinants, with this study we assessed the potential link between metabolic implications and two distinct types of allergies. Methods: Using cross-sectional data from the German FoCus cohort, *n* = 385 allergy cases, either hay fever (=type I allergy, *n* = 183) or contact allergy (=type IV allergy, *n* = 202) were compared to age- and sex-matched healthy control subjects (1:1 ratio, in total *n* = 770) regarding their metabolic phenotype, diet, physical activity, sleep, gut microbial composition, and serum metabolite profile using suitable BMI-adjusted models. Results: Obesity and metabolic alterations were found significantly more prevalent in subjects with allergies. In fact, this relation was more pronounced in contact allergy than hay fever. Subsequent BMI-adjusted analysis reveals particular importance of co-occurring hyperlipidaemia for both allergy types. For contact allergy, we revealed a strong association to the dietary intake of poly-unsaturated fatty acids, particularly α-linolenic acid, as well as the enrichment of the corresponding metabolic pathway. For hay fever, there were no major associations to the diet but to a lower physical activity level, shorter duration of sleep, and an altered gut microbial composition. Finally, genetic predisposition for hyperlipidaemia was associated to both contact allergy and hay fever. Conclusions: Reflected by higher allergy prevalence, our findings indicate an impaired immune response in obesity and hyperlipidaemia, which is differentially regulated in type I and type IV allergies by an unfavourable lifestyle constellation and subsequent microbial and metabolic dysfunctions.

## 1. Introduction

The prevalence of allergies has been increasing in the last few decades. According to the findings of the Study of Healthy Adults in Germany (orig. title: “Studie zur Gesundheit Erwachsener in Deutschland”) in 2008–2011, approximately 20% of German adults had at least one allergy, with women being more likely to be affected than men [1]. Furthermore, the World Health Organization (WHO) estimated that over 1.9 billion adults were overweight, with over 650 million obese [2]. Since both obesity and allergies are highly influenced by environmental determinants, significant studies have been conducted on both. Besides that, obesity has also been known as a major health issue not only related to modulators of the innate but also the adaptive immune response. On that note, previous studies have reported a link between obesity and an increased prevalence of different allergic disorders including asthma [3], food [4], and drug allergies [5,6]. While these previous studies have reliably reported an increased prevalence of allergies in overweight and obese people, the causes of this association are not very well understood yet.

The aim of our study is therefore a comprehensive analysis of potential contributing factors for the development of allergies covering various lifestyle-associated determinants, including the diet, physical activities, sleep and smoking habits, the gut microbial composition, serum metabolite profiles, as well as selected genetic variations. In order to account for possible distinctions between different types of allergies, we will mostly be conducting separate analyses for differentially regulated allergy types. Here, distinction will be made between the presence of either hay fever representative for IgE-mediated type I or contact allergy representative for T cell-mediated type IV allergic hypersensitivities.

For this exploratory approach, we made use of the extensively characterised German population-based FoCus cohort (*n* = 1800). Information on the occurrence of allergies were used to define a subset of *n* = 770 subjects equally distributed into allergy cases (*n* = 202 stratified by contact allergy, *n* = 183 stratified by hay fever) and allergy-free healthy control subjects. Using a case-control study design which reduces the possible confounding effects of participants’ age and biological sex, we assessed questionnaire-based health data, obesity-associated anthropometric measures, as well as metabolic and inflammatory serum markers. Furthermore, nutritional information collected using a valid food frequency questionnaire (FFQ), microbial 16S rRNA sequencing data from faecal samples, untargeted FT-ICR-MS serum metabolomics, and microarray-based data on genetic variation were available for analysis.

## 2. Material and Methods

### 2.1. Study Design and Population

For a comprehensive assessment of allergy occurrence in relation to obesity, we made use of the German population-based Food Chain Plus (FoCus) cohort. Data collection took place between 2011 and 2014 including a total of 1800 adults of different ages. Extensive anthropometric data, metabolic and inflammatory parameters measured in serum, intestinal microbiome 16S rRNA sequencing data, information about dietary habits, physical activities, sleeping duration, and smoking status, as well as genetic variation have been collected and are available for analysis.

**Figure 1:**
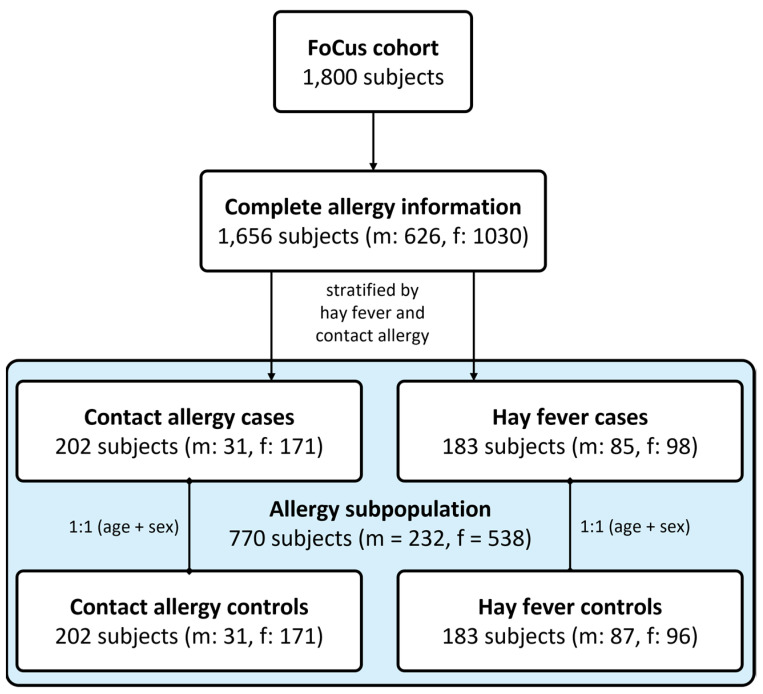
Flowchart of the defined study populations. Using the cross-sectional population-based FoCus cohort (total *n* = 1800), we selected subjects with complete allergy information (*n* = 1656) for an overall allergy analysis. We then stratified these subjects by the presence of either contact allergy (*n* = 202) or hay fever (*n* = 183) and matched them to their nearest healthy allergy-free controls (*n* = 770). Abbreviations: m = male, f = female.

Complete allergy information was available for *n* = 1674 subjects. As a first step, general allergy occurrence, defined by the presence of at least one allergy of any kind, has been assessed regarding potential associations with participants’ age and biological sex. Subsequently, a case-control study design was selected to remove the possible confounding effects of the stated variables. Furthermore, stratification of participants was conducted in order to account for the differing mediation of type I (IgE-mediated) and type IV (T cell-mediated) allergies based on the most present kind of both types (hay fever vs. contact allergy). By that, *n* = 183 with hay fever and *n* = 202 participants with contact allergy were extracted for detailed analyses. Healthy, allergy-free control subjects were identified by the absence of any allergy as well as inflammatory, cardiovascular, metabolic, and pulmonary chronic diseases and matched to the nearest fitting allergy case regarding the biological sex and age in a 1:1 ratio (see Figure 1).

### 2.2. Allergy Information

Using a medical questionnaire, information on participant’s anthropometric characteristics, lifestyle, and health status were obtained. The questionnaire consists of 58 questions in total, including questions regarding present allergies. Participants with a contact allergy were recognized through the question “Have you ever been diagnosed with a contact allergy (itching “rash” at contact points caused by jewelry, perfumes, skin care products, cosmetics, textiles, work materials, ointments, or other substances) by a doctor?”. Hay fever was surveyed through the question “Have you ever been diagnosed with hay fever (allergic rhinitis) by a doctor?”.

### 2.3. Anthropometric Measurements

Patients were weighed on digital scales in light clothing without shoes. Using a stadiometer, height was measured. Using measuring tape, waist and hip circumferences were measured. All physical examinations were performed in duplicates by trained medical staff. For data analyses, mean values were formed. Body mass index (BMI) and waist-to-hip ratio (WHR) were calculated using respective established formula.

### 2.4. Biochemical Analysis

Blood samples were collected by venepuncture after an overnight fast. In the central laboratory of the University Medical Center in Kiel, clinical metabolic and inflammatory markers were analysed from the collected blood samples, including *C*-reactive protein (CRP), interleukin-6 (IL-6), fasting glucose, fasting insulin, and triglycerides. Fasting glucose and fasting insulin were then used to calculate Homeostatic Model Assessment of Insulin Resistance (HOMA-IR/insulin sensitivity).

### 2.5. Taste Perception

Bitter and saline taste perception testing has been performed by all included subjects. In brief, after neutralisation with water, 0.02 L of bitter solution (6-n-propylthiouracil, 273 mg/500 mL) was put in the mouth to subsequently mark the perceived taste intensity immediately and intuitively on a general labelled magnitude scale (0–300). This procedure was repeated for the second taste solution, containing the tasting agent sodium chloride (NaCl, 29 g/500 mL).

### 2.6. Dietary Assessment

Dietary intake was assessed using a valid semi-quantitative 12-month retrospective food frequency questionnaire (FFQ, European Investigation into Cancer and Nutrition protocol, German Institute of Human Nutrition, Potsdam) [7]. Validation of the FFQ has been previously described in detail by Nöthlings et al. [8]. Supplementary, accuracy was internally validated via unannounced 24 h-dietary recall in 10% of subjects.

The FFQ consists of questions regarding frequency of consumption and average portion sizes of 134 foods. Subsequent analysis was realized using “EPICsoft” resulting in the daily intake of micro- and macronutrients [9]. Furthermore, the FFQ also includes information on participant’s physical activity level and duration of sleep.

### 2.7. Gut Microbiome Analysis

For the evaluation of gut microbiome, stool samples were collected. DNA from stool samples was extracted using the QIAamp DNA stool mini kit automated on the QIAcube. Analysis was done using R and RStudio. “phyloseq”, “microbiome”, and “vegan” were among the packages used in R. To measure gut microbiome diversity, several indices were used. Intra-individual variance (α-diversity) was measured using two indices: (1) Species Richness and (2) Shannon index. Inter-individual variance (β-diversity) was measured using two approaches: (1) Bray–Curtis dissimilarity approach and (2) binary Jaccard approach on presence and absence of core microbes (=amplicon sequencing variants (ASVs) present in >40% of participants with a relative abundance of ≥0.5%) [10].

### 2.8. Serum Metabolomics Analysis

For a subset of participants (*n* = 404), untargeted detection data of 955 metabolites from serum samples were available for analysis. Procedures of sample collection and processing have recently been described in detail by Henneke et al. [11]. In brief, serum samples were extracted by a modified SIMPLEX approach [12] of which a lipophilic methyl-tert-butyl ether (MTBE) phase, a hydrophilic methanol-water phase, and a protein pellet were obtained, dried under vacuum, and resuspended with specific solvents: isopropanol/chloroform (3/1, *v*/*v*) with 0.1% acetic acid (lipophilic); and water/methanol (50/50, *v*/*v*) with 0.1% acetic acid (hydrophilic), both supplemented by specific internal standard mixture. Mass spectrometry was conducted using an FT-ICR-MS in flow-injection mode being facilitated by HPLC autosampler. The eluent was water/methanol (50/50, *v*/*v*) with 0.1% acetic acid (hydrophilic) and isopropanol/chloroform (3/1, *v*/*v*) with 0.1% acetic acid (lipophilic). All samples were ionized with an electrospray ionization source. Different methods were used, each optimized to the respective detection range (in total, the range was from 65 to 3000 *m*/*z*). Evaluation of mass features was conducted with DataAnalysis 5.0 and MetaboScape 4.0.1. (Bruker Corporation, Bremen, Germany). Sum formulas were calculated and the seven golden rules of Kind and Fiehn [13] were applied.

Based on the results of mass spectrometry metabolite analysis, which we realised using two-part hurdle models of binomial logistic regression and negative binomial logistic regression for the zero-inflated detection data, we performed a pathway analysis in MetaboAnalyst 5.0 aiming to identify common pathways among selected metabolites (enrichment) and their impact to the respective pathways (topological). Here, all metabolites that remained significant after an FDR correction of *p*-values in the count part of the hurdle model were included.

### 2.9. Genetic Variation Analysis

Depending on availability of whole blood samples, participants were genotyped using the Iscan Immunochip Opticall and the Iscan Omniexpress Exome Chip (*n* = 671). Quality control and preprocessing of genetic data have previously been described elsewhere [14]. Quality control of genetic data was done in PLINK v.1.9. A total of 19 SNPs in the low-density lipoprotein receptor (*LDL-R*) gene (chr19, 11.20–11.24 Mb), 70 SNPs in the very-low-density lipoprotein receptor (*VLDL-R*) gene (chr9, 26.22–26.53 Mb), 14 SNPs in the proprotein convertase subtilisin/kexin type 9 (*PCSK9*) gene (chr1, 55.50–55.52 Mb), and 15 SNPs in the apolipoprotein B (*apoB*) gene (chr2, 21.22–21.26 Mb) passed quality control and filtering. BMI- and hyperlipidaemia-adjusted logistic regression was used to test for associations between genetic variations and allergy occurrence. For all analyses, the SNP wildtype was defined as the reference group.

### 2.10. Statistical Evaluation

Statistical analysis and visualization were realized using R (R Core Team, 2022) and R Studio Version 1.3.1093 (Rstudio., Inc., Boston, MA, USA) with case-control matching being performed to the nearest match using “MatchIt” package [15].

Continuous variables were tested for normal distribution with Shapiro–Wilk test. Normally distributed data are displayed as mean ± standard deviation, and non-normally distributed data as mean (interquartile range). Categorical variables are expressed as counts and corresponding percentages. Univariate compassion of categorical variables between two independent groups was performed using χ^2^ Test. Univariate comparison of continuous variables between two independent groups was performed using Mann–Whitney U Test. For allergy contribution assessment, BMI-adjusted logistic regression models were used subsequently. In order to account for the zero-inflated character of microbial count and metabolite detection data, we performed BMI-adjusted two-part hurdle models for the assessment of these data in relation to allergy occurrence. The first part uses a logistic regression comparing values = 0 with values ≠ 0 (zero-part). The second part uses a negative binomial logistic regression by truncating zero values hence only including continuous values >0 (count-part). To prevent the description of false positive results, hurdle models (>500 independent variables per dataset) were supplemented by the correction of *p*-values using the FDR-method. For all analyses, statistical level was at alpha < 0.05.

## 3. Results

Using comprehensive cross-sectional cohort data from our German population-based FoCus cohort, we aim to precisely investigate the occurrence of allergies in relation to obesity at metabolic, inflammatory, dietary, microbiome, metabolome, and genetic level.

### 3.1. Overall Allergy Prevalence in the FoCus Cohort

As a first analysis, we assessed the overall allergy prevalence within the *n* = 1656 subjects who provided complete questionnaire-based allergy information.

**Figure 2:**
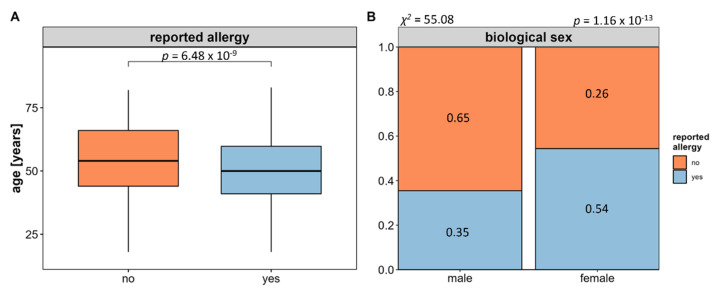
Overall allergy occurrence in relation to age and biological sex. Using questionnaire-based information from the FoCus cohort on any reported allergy reveals a significant relation to both age and participants’ biological sex. Allergies are associated to a younger age (**A**) statistical significance tested using Mann–Whitney U Test) and are more prevalent in females (**B**) statistical significance tested using χ^2^ Test).

Of these, 782 reported at least one type of allergy reflecting an overall allergy prevalence of 47.22% for the FoCus cohort. Comparing the age of participants with and without allergy, it appears that allergies are associated with a younger age (see Figure 2A). Regarding the biological sex, the proportion of women affected by allergy is significantly higher than that of men (see Figure 2B).

In order to account for possible confounding effects of the above associations, we decided to perform all the following analyses in an age- and sex-matched case-control manner. To further account for known regulatory differences of different allergy types, we stratified participants according to either the presence of contact allergy as the most prevalent T cell-mediated type IV allergy or hay fever as the most prevalent IgE-mediated type I allergy (also see Section 2.1) resulting in an extracted subpopulation of 770 subjects equally distributed into allergy cases and healthy, allergy-free control subjects. Clinical characteristics, including anthropometric measures, metabolic and inflammatory serum markers, as well as metabolic comorbidities, are provided in Table 1.

### 3.2. Assessment of Allergy Prevalence in Obesity

**Figure 3:**
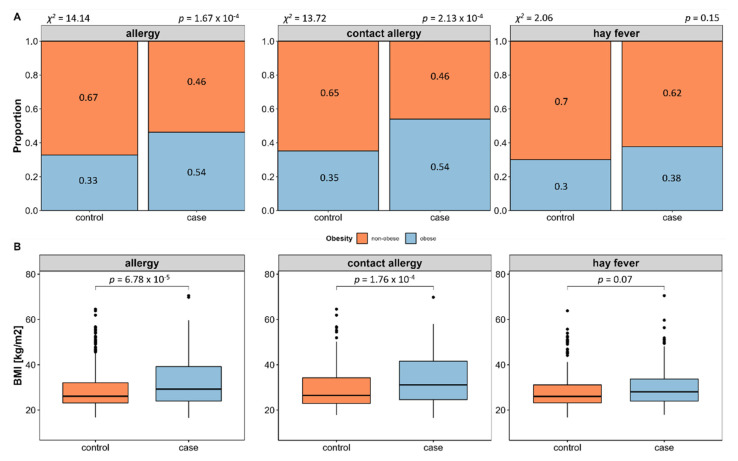
Allergy prevalence in relation to obesity. Case-control comparison of age- and sex-matched subjects with and without allergies reveals significantly higher obesity proportion and BMI in allergy cases. Subsequent individual evaluation of contact allergy and hay fever shows that obesity is actually more prevalent in contact allergy, while this is not the case in hay fever (**A**) statistical significance evaluated using χ^2^ Test). These results are in line with a significant higher BMI of contact allergy but not hay fever subjects (**B**) statistical significance tested by Mann–Whitney U Test).

We evaluated allergy prevalence in relation to obesity and associated measures. This revealed a significant higher prevalence of allergies in obese (BMI ≥ 30) compared to non-obese (BMI < 30) subjects (58.55%, *p* = 1.67 × 10^−4^, see Figure 3A), which is in line with a significant higher BMI (*p* = 6.78 × 10^−5^, see Figure 3B) and waist-to-hip ratio in allergy cases (*p* = 1.28 × 10^−2^). Subsequent individual evaluation of contact allergy and hay fever shows that obesity is in fact more prevalent in contact allergy, while this is not the case in hay fever.

Because of the differing mediation of contact allergy and hay fever and the disparity regarding obesity association, we decided to perform all further analyses separately for contact allergy and hay fever. Furthermore, BMI was included as an additional confounder in all the following analyses to assess whether effects may rather be caused by obesity than allergy.

### 3.3. Impact of Metabolic Alterations on Allergy Occurrence

We next evaluated the impact of metabolic alterations on the occurrence of the two allergy types. Results of BMI-adjusted binomial logistic regression are presented in Table 2. Here, changes in the lipid metabolism, represented by elevated serum cholesterol levels and the presence of manifest hyperlipidaemia, contribute to an increased occurrence of both contact allergy (hyperlipidaemia: OR = 1.62, *p* = 3.57 × 10^−2^) and hay fever (hyperlipidaemia: OR = 1.49, *p* = 4.15 × 10^−2^; cholesterol: OR = 1.63, *p* = 3.05 × 10^−2^). Apart from that, neither impairment of glucose metabolism, nor inflammatory activity, nor other comorbidities had direct effects on the presence of contact allergy or hay fever.

### 3.4. Impact of Different Lifestyle Determinants on Allergy Occurrence

In the next step, we assessed different lifestyle determinants regarding their contribution to allergy occurrence comprising detailed information on dietary intake, physical activities, duration of sleep, and smoking habits.

#### 3.4.1. Dietary Intake in Relation to Allergy Occurrence

For the evaluation of the impact of participant’s diet and to identify potential dietary compounds that may be associated to the occurrence of allergies, we assessed dietary information that was collected using 12-months retrospective FFQ. Selected characteristics of the subjects’ diet are shown in Table 3.

BMI-adjusted binomial logistic regression of dietary information shows significant contribution of different dietary compounds to contact allergy and hay fever risk. Analysing four main dietary compounds (carbohydrates, fats, proteins, and fibres), contact allergies were significantly related to a reduced intake of dietary fats (OR = 0.96, *p* = 2.73 × 10^−2^). In more detail, it becomes clear that this effect was mostly due to long-chain fatty acids (LCFA, OR = 0.96, *p* = 2.63 × 10^−2^) of poly-unsaturated nature (PUFA: OR = 0.88, *p* = 4.35 × 10^−2^; linolenic acid: OR = 0.94, *p* = 1.78 × 10^−2^) which is plausible regarding the previously identified association to alterations of the lipid metabolism. Considering mineral and vitamin intake, iodine (OR = 6246.45, *p* = 5.37 × 10^−3^) and fluorine (OR = 2.49, *p* = 2.65 × 10^−4^) supply were significantly increased, while vitamin E (OR = 0.93, *p* = 3.43 × 10^−2^) and niacin (OR = 0.91, *p* = 2.2 × 10^−3^) supply were significantly increased in contact allergy. We were not able to identify a contribution of any of the main nutrient classes regarding hay fever occurrence. Still, detailed micronutrient assessment reveals low calcium (OR = 0.23, *p* = 3.17 × 10^−3^) and lactose (OR = 0.96, *p* = 8.87 × 10^−3^) supply, while a high iron intake was observed to be associated to higher hay fever rates (OR = 1.17, *p* = 2.28 × 10^−2^).

#### 3.4.2. Taste Perception in Relation to Allergy Occurrence

Due to the allergy specific symptoms of swollen mucosal membranes, mainly in the nose and mouth, we were interested in potential associations to an impairment of taste perception. However, neither contact allergy nor hay fever were associated with an altered bitter or saline taste perception.

#### 3.4.3. Physical Activity, Sleep, and Smoking Habits in Relation to Allergy Occurrence

In addition to the information on subjects’ diet, data on physical activities, duration of sleep, and smoking habits were assessed in regard to their potential contribution to allergy risk. Characterization of these determinants is displayed in Table 4.

BMI-adjusted logistic regression reveals protective properties of vigorous physical activity (OR = 0.94, *p* = 6.76 × 10^−3^) against the development of hay fever. Further assessment reveals that this effect is specifically associated to outdoor cycling activity (OR = 0.86, *p* = 1.29 × 10^−3^). A sufficient duration of sleep is also associated to reduced hay fever occurrence (OR = 0.75, *p* = 2.32 × 10^−3^).

Of interest, we were not able to identify any effect for the tested variables with contact allergy occurrence.

### 3.5. Impact of the Gut Microbial Composition on Allergy Occurrence

Since obesity and an associated lifestyle are known to be strongly linked to the composition of the gut microbiome, we next investigated the potential role of the intestinal microbiota in the development of allergic diseases. For this purpose, 16 S rRNA sequencing data of the gut microbiome was used to assess the composition of a core microbiome, a variety of indices of microbial diversity, and potential candidate microbial strains.

**Figure 4:**
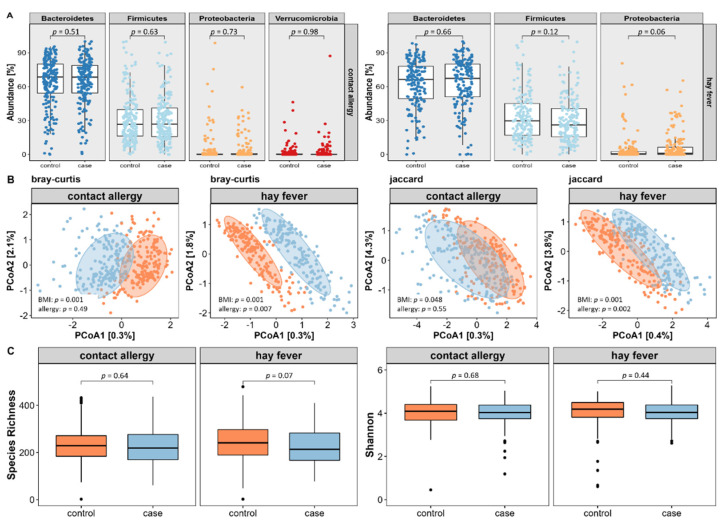
Relation of the gut microbial composition in relation to the occurrence of contact allergy and hay fever. We assessed the composition of a core microbiome and a variety of indices of microbial diversity. Composition of the core microbiome on phylum levels proposes no major differences between the two types of allergies and their respective controls (**A**). When further assessing inter-individual differences, hay fever displayed significant microbial variance using both Bray–Curtis and Jaccard indices (**B**). Regarding intra-individual variance, there were no distinct differences (**C**). All displayed *p*-values refer to BMI-adjusted calculations.

First, we extracted core microbes by selecting amplicon sequence variants (ASVs) that were present in at least 40% of subjects with a relative abundance of ≥0.5%. By that, we identified 47 core microbial strains (=amplicon sequencing variants (ASVs) present in >40% of participants with a relative abundance of ≥0.5%) for the contact allergy and 50 for the hay fever subpopulation. Using BMI-adjusted logistic regression of relative abundances on phylum level, we did not reveal any major differences in neither contact allergy nor hay fever compared to their respective control subjects (see Figure 4A).

For the evaluation of microbial diversity, we assessed both inter- and intra-individual variance using appropriate indices. As displayed in Figure 4B, inter-individual variance, also referred to as β-diversity, did not differ between contact allergy cases and controls using PERMANOVA based on the Bray–Curtis dissimilarities index (*p* = 0.49) or on the Jaccard presence/absence index of core microbes (*p* = 0.55). In contrast, hay fever reveals significant differences of both Bray–Curtis (*p* = 0.005) and Jaccard (*p* = 0.003) index.

Regarding intra-individual microbial variation, also known as α-diversity, no differences were found between cases and controls assessing the species richness or Shannon index (see Figure 4C).

We additionally performed BMI-adjusted hurdle models of microbial counts not restricted to core microbes (16,385 ASVs, FDR-corrected) in order to identify potential single microbial candidates. By that, we identified 411 ASVs that contributed and were altered in contact allergy, of which 282 ASVs were taxonomically classified up to genus level. For hay fever, we were able to identify a total of 393 significant ASVs, of which 242 ASVs were taxonomically classified up to genus level. Of note, significant associations were exclusively seen in the zero-truncated negative binomial regression (count) part of the hurdle model.

**Figure 5:**
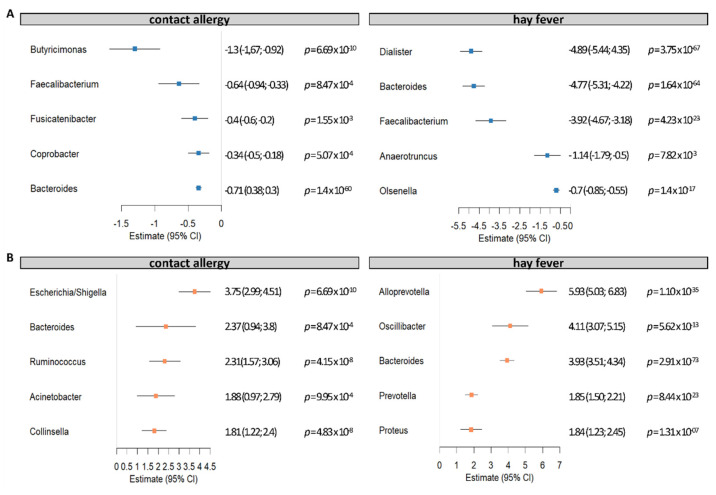
Impact of selected candidate bacteria on the occurrence of contact allergy and hay fever. Using a BMI-adjusted two-part hurdle model, we assessed a total of 16,385 ASVs in relation to allergy occurrence. Significant associations were exclusively seen in the zero-truncated negative binomial regression part of the hurdle model. We found a total of 282 taxonomically well characterized ASVs significantly contributing to contact allergy, of which 71 were reduced (estimate < 0) (5 highest estimates, (**A**), and 211 which were increased in contact allergy (estimate > 0) (5 highest estimates, (**B**). For hay fever, we identified a total of 242 well characterized ASVs, of which 65 were reduced (5 highest estimates, (**A**) and 177 which were increased in hay fever (2–6 highest estimates, (**B**). Notably, the most altered ASV of the genus *Clostridium cluster XlVb* in hay fever is not displayed due to a very high estimate of 29.49 (15.43; 35.55, *p* = 2.02 × 10^−5^).

The strongest contributors, both decreased (five lowest estimates) and increased (five highest estimates) in allergies, are displayed in Figure 5. We identified ASVs of the genera *Butyricimonas*, *Faecalibacterium*, *Fusicatenibacter*, *Coprobacter*, and *Bacteroides* reduced in contact allergy. By contrast, ASVs of the genera *Escherichia/Shigella*, *Bacteroides*, *Ruminococcus*, *Acinetobacter*, and *Collinsella* were increased in contact allergy. *Dialister*, *Bacteroides*, *Faecalibacterium*, *Anaerotruncus*, and *Olsenella* were reduced in hay fever, while ASVs of the genera *Clostridium cluster XlVb*, *Alloprevotella*, *Oscillibacter*, *Bacteroides*, *Prevotella*, and *Proteus* were more abundant in hay fever.

### 3.6. Impact of Serum Metabolites on Allergy Occurrence

FT-ICR-MS metabolomics data were available for 403 subjects (118 with contact allergy, 106 with hay fever, and 224 control subjects) of the study population. Using BMI-adjusted two-part hurdle models, we assessed a total of 955 detected metabolites. As for the microbiome analysis, significant associations were only seen in the zero-truncated negative binomial regression (count) part of the hurdle model. Here, 118 metabolites were nominally associated with the presence of contact allergy, of which 23 remained significant after FDR-adjustment for multiple comparisons. A total of 124 metabolites were nominally associated with the presence of hay fever, of which 35 remained significant after FDR-adjustment for multiple comparisons.

A subsequent pathway analysis (enrichment and topological) of the significant metabolites resulted in three nominally significant enriched pathways for contact allergies and one pathway for hay fever that are displayed in Table 5.

### 3.7. Impact of Genetic Predisposition on Allergy Occurrence

**Figure 6:**
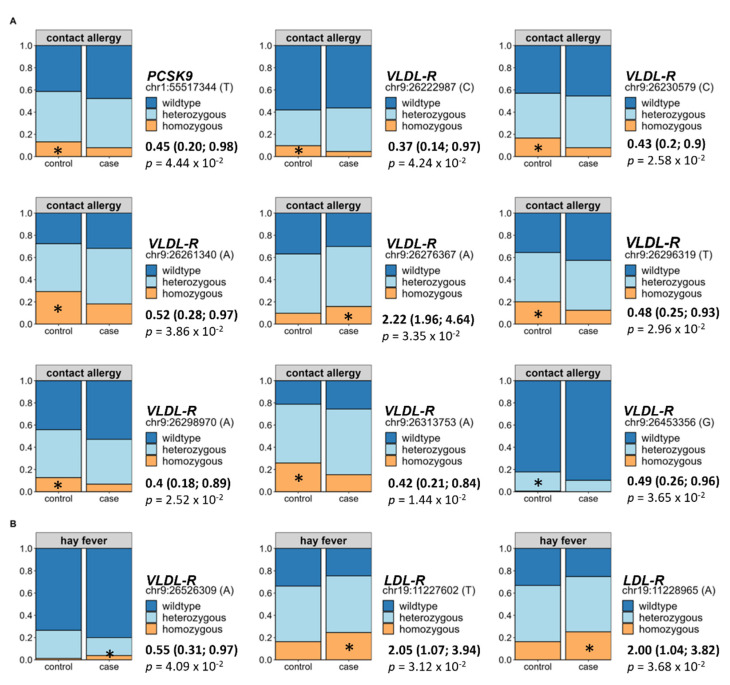
Impact of selected hyperlipidaemia-associated single nucleotide polymorphisms (SNPs) on the development of contact allergy and hay fever. We assessed selected hyperlipidaemia-associated genetic variations in relation to allergy occurrence using BMI- and hyperlipidaemia-adjusted binomial logistic regression (odds ratios + confidence intervals, *p*-values, and significance levels (*: *p* < 0.05) are displayed corresponding to each analysis). Out of the 118 tested SNPs originating from either the *PCSK9* (*n* = 14), *LDL-R* (*n* = 19), *VLDL-R* (*n* = 70), or *apoB* (*n* = 15) gene, 9 SNPs were directly associated to an increased prevalence of contact allergies. In detail, eight of the identified SNPs were variations in the *VLDL-R* and one in the *PCSK9* gene (**A**). Regarding hay fever, two SNPs of the *LDL-*R and one SNP of the *LDL-R* showed a direct association to allergy occurrence (**B**). For all analyses, the wildtype was set as reference group.

Finally, since we identified direct effects of alterations in the lipid metabolism on the prevalence of both allergy types, we assessed selected hyperlipidaemia-associated variations of the *LDL-R*, *VLDL-R*, *apoB*, and *PCSK9* gene in relation to allergy occurrence using BMI- and hyperlipidaemia-adjusted binomial logistic regression. Out of the 118 tested SNPs, 9 were directly associated to an increased prevalence of contact allergy. In detail, eight of these originated from the *VLDL-R* and one from the *PCSK9* gene (see Figure 6A). Regarding hay fever, two SNPs of the *LDL-*R and one SNP of the *LDL-R* showed a direct association to allergy occurrence (see Figure 6B). For all analyses, the wildtype was set as the reference group.

## 4. Discussion

The prevalence of allergies has, similar to that of metabolic diseases, rapidly increased during the past few decades [1]. Moreover, both allergies and obesity are strongly influenced by lifestyle determinants, especially the diet. These parallels propose a relation between these two diseases, which in fact has been proven in previous studies on asthma [3], food [4], and drug [5,6] allergies. However, despite the evidence, causes of this association are not very well understood yet. With our study, we aim to expand current knowledge about the regulation of allergies in the context of metabolic comorbidities, especially obesity. We used data from the German population-based FoCus cohort enabling us to identify distinctions between allergy-affected and non-affected participants on metabolic, inflammatory, lifestyle, microbiome, metabolome, and genetic level. Further information on the present allergy type also ensured the differentiation between IgE-mediated type I and T cell-mediated type IV allergies. We identified a positive relation between the presence of allergies, obesity, and hyperlipidaemia different in type I and type IV allergies.

With our data, we can display a co-occurrence of allergies and obesity as well as a positive association with related anthropometric and metabolic measures. This relation is more pronounced in contact allergies than in hay fever, supporting already known differing regulatory mechanisms. The strong association of BMI and contact allergies is in line with the results of Lusi et al., who found an increased prevalence of nickel allergy in overweight participants [16]. An Italian case-control study including 1128 participants also reported an increased BMI and further identified alterations in the glucose metabolism and inflammatory activity in nickel allergy participants [2]. These alterations are also present in our study population; however, considering participants’ BMI as a confounding factor proposes that these are rather associated to the presence of obesity than directly to the contact allergy. Additionally, Watanabe et al. were also able to display significant alterations in the body composition of increased fat and decreased lean mass with the presence of nickel allergy [17], which is complementing the significant higher waist-to-hip ratio as a marker of body composition, particularly body fat distribution within our study. These findings might propose immunological properties of the adipose tissue in the relation of contact allergies and obesity. However, for a precise conclusion on this relation, further studies are needed.

Above that, we were able to identify a direct contribution of alterations in the lipid metabolism to both the risk for contact allergy and hay fever. This is of interest since previous studies have not yet allowed for a clear valuation of this association mostly due to the lacking assessment of potential influencing factors [18]. Consistent with a higher proportion of hyperlipidaemia in contact allergy in comparison to the respective control group, we found a significant association with the participants’ dietary fat intake. While a Korean study with 3040 participants reported an association of allergic rhinitis with a high-fat and low-carbohydrate diet proposing a positive association of dietary fat intake and allergy occurrence overall [19], another study by Trak-Fellermeier et al. revealed allergic sensitization in females with imbalanced fat intake in particular [20]. On micronutrient level, an investigation of dietary α-linolenic acid-rich linseed oil showed dampening effects of allergic symptoms by 15-hydroxyeicosapentaenoic acid, a metabolite of eicosapentaenoic acid (EPA), which was eosinophile-dependently produced in a mouse study [21]. Another study by Kunisava et al. identified 17,18-epoxyeicosatetraenoic acid, another metabolite of EPA, as an anti-allergic lipid metabolite in the same mouse model [22]. These findings postulate the generation of lipid anti-allergic metabolic mediators out of dietary omega-3 fatty acids for the counteraction of allergic reactions. This is of particular interest since we did not only find poly-unsaturated fatty acids and specifically α-linolenic acid on the dietary, but also α-linolenic und glycerophospholipid metabolism on the metabolome level associated to contact allergy occurrence. Hence, with our data we can clearly underline the important role of a favourable, omega-PUFA-rich dietary fat constitution and involvement of lipid metabolites for the prevention or improvement of contact allergies.

Even though hay fever is equally associated to the presence of hyperlipidaemia and cholesterol serum levels, we were not able to identify major differences in the dietary composition on macronutrient level proposing other regulatory factors. On that note, hay fever was negatively associated to the duration of sleep which is consistent with results from a US-based cross-sectional study that has also reported an association between hay fever and sleep parameters, such as insomnia and sleep apnoea [23]. However, to the best of our knowledge, there is no further evidence on this relation yet. Hay fever was associated to lower weekly rates of sports activity compared to their respective controls. There has been previous research that revealed that elite athletes had an increased risk for asthma and allergy [24]. An explanation for this was given by their frequent exposure to pollen allergens during exercise in spring, summer, and winter, which might have led to bronchial hyper-responsiveness [25]. Granted that the protective properties observed within our study was not constituted of athletes, there might be a dose- and intensity-dependent relation between sports and allergy risk possibly further affected by the performance of either indoor or outdoor activities. Nevertheless, a short duration of sleep and the lack of physical activity are strongly associated to multiple unfavourable health outcomes and specifically conveyed by alterations of the lipid metabolism [26,27]. Regarding the differences between contact allergy and hay fever, our findings indicate that they both are mediated by the impairments of the lipid metabolism but might be regulated by different lifestyle determinants of either dietary fat, physical inactivity, or an insufficient amount of sleep.

Apart from the regulation by lifestyle determinants, alterations in the lipid metabolism can also be caused by a genetic predisposition. Here, variations in the *PCSK9*, *apoB*, *LDL-R*, and *VLDL-R* gene are possible reasons for dysregulated blood lipids. Of interest, we were able to identify mild associations between hyperlipidaemia-associated genetic single nucleotide polymorphisms (SNPs) of the stated genetic loci to both contact allergies and hay fever. While contact allergy showed impairment in the *PCSK9* and *VLDL-R* SNPs, hay fever displayed alterations in the *LDL-R* and *VLDL-R* receptor considering both the BMI and the presence of hyperlipidaemia as confounding factors. These findings indicate that aside from the lifestyle, genetic predisposition of metabolic implications might shape the risk of allergies in obesity and hyperlipidaemia.

The gut microbiome also plays a key role in the development of chronic conditions for numerous reasons. As a result, one explanatory approach for the increasing allergy prevalence is centred around the gut microbial changes caused by the rapid changes in diet and general lifestyle patterns over the last century [28]. For a functioning immune system, a sensitive balance between commensal and pathogenic microbial strains is mandatory. However, not only the microbiome as a whole, but also the increased occurrence of individual microbial strains can unbalance the immune function or even cause infection [29]. To assess if this holds true in our study, we performed broad microbial analysis. By that, we were able to identify differences in the inter-individual microbial variance as well as a clear trend towards impaired microbial intra-individual diversity between hay fever and their respective control subjects. This finding is in line with the results of Watts et al., who presented altered microbial composition reflected in reduced diversity and a shift in microbes on phylum, genus, and species level [30]. Notably, a high microbial diversity majorly contributes to a sufficient defense against exogenous pathogens [31]. We were not able to identify major differences in the microbial composition of contact allergies. Still, alterations were present in the abundance of single microbial strains that may contribute to contact allergy risk. Of note, several butyrate-producing genera (*Butyricimonas, Faecalibacterium, Fusicatenibacter,* *Dialister*, *Anaerotruncus*) were reduced in allergic individuals. Butyrate has been associated with the induction of regulatory T cells which may counteract allergic Th2 induction [32]. Above the diverse immune-regulatory properties of the microbiota, the intestinal system also represents an important barrier against environmental, and especially dietary pathogens. Obesity and an excessive dietary fat intake in particular can impact this barrier function and by that directly contribute to an imbalanced immune response by the initiation of local and systemic inflammation [33]. On that note, there is reliable evidence on increased gut permeability in subjects with allergic diseases [34,35] which might also be the case in contact allergy within our study and could be an explanation for the strong effects seen related to the obese phenotype and dietary pattern.

### Limitations

With our study, we can only shed light on the associations seen with the co-occurrence of contact allergy and obesity but are not able to conclude on the exact causality of this (1) because we do not have information on the order of disease onset and (2) further information especially regarding the immune function, e.g., cytokine profiles, would be necessary for a precise biochemical interpretation.

## 5. Conclusions

**Figure 7:**
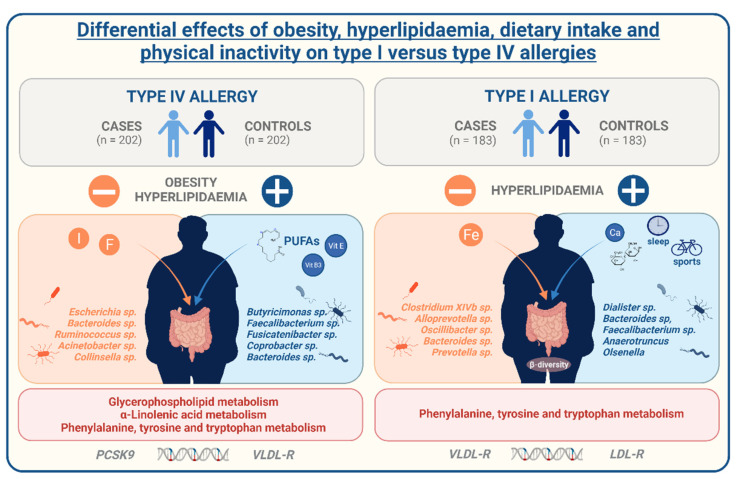
Differential effects of obesity, hyperlipidaemia, dietary intake, and physical inactivity on type I versus type IV allergies. Our data clearly indicate the co-occurrence of allergies, obesity, and hyperlipidaemia differentially mediated depending on the type of present allergy. While contact allergies are strongly associated to obesity coupled to the presence of hyperlipidaemia, an imbalance in dietary fat intake, and lipid metabolic pathways, hay fever is stronger associated to alterations of the lipid metabolism than obesity which is predominantly mediated by reduced levels of physical activity, decreased duration of sleep, and altered gut microbial composition. Abbreviations: I = iodine; F = fluorine; PUFAs = poly-unsaturated fatty acids; Vit = vitamin; Fe = iron; Ca = calcium; PCSK9 = proprotein convertase subtilisin/kexin type 9; VLDL-R = very low-density lipoprotein receptor; LDL-R = low-density lipoprotein receptor.

Our data clearly indicate the co-occurrence of allergies, obesity, and hyperlipidaemia differentially mediated depending on the type of present allergy (see Figure 7). While contact allergies are strongly linked to obesity coupled to the presence of hyperlipidaemia, an imbalance in dietary fat intake, and lipid metabolic pathways, hay fever is stronger associated with alterations of the lipid metabolism than obesity which is predominantly mediated by reduced levels of physical activity, decreased duration of sleep, and altered gut microbial composition. Taken together, our results support the complex effects of exogenous lifestyle determinants and subsequent metabolic implication on the adapted immune response resulting in higher rates of both IgE and T cell mediated allergies. For the effective counteraction of both these metabolic and immunological disorders, their complex interaction should be the subject of further studies, especially on a functional level.

## Figures and Tables

**Table 1:** Clinical characterization of the study population.

	Contact Allergy			Hay Fever		
	Cases	Controls	*p*	Cases	Controls	*p*
subjects, *n* (%) ^a^	202 (50)	202 (50)	1.0	183 (50)	183 (50)	1.0
female sex, *n* (%) ^a^	171 (84.65)	171 (84.65)	1.0	83 (45.36)	96 (52.46)	0.75
age, years ^b^	50 (44; 61)	50 (44; 61)	0.99	50 (44; 61)	50 (44; 61)	0.99
anthropometric measures						
height, cm ^b^	169 (163; 174.88)	169 (164; 175)	0.65	172.89 ± 8.97	175.05 ± 9.27	0.04
weight, kg ^b^	88.08 (70.7; 118.36)	75.57 (65.95; 96.34)	1.88 × 10^−4^	83.55 (71.83; 103.1)	80.9 (69.97; 98.45)	0.42
**BMI, kg/m^2 b^**	**31.1 (24.6; 41.58)**	**26.46 (22.93; 34.27)**	**1.76 × 10^−4^**	**28.05 (23.94; 33.72)**	**26.01 (23.14; 31.14)**	**0.07**
waist-to-hip ratio ^b^	0.87 (0.81; 0.92)	0.84 (0.78; 0.91)	1.03 × 10^−2^	0.89 ± 0.10	0.88 ± 0.09	0.29
metabolic markers						
glucose, mg/dL ^b^	95 (89; 104)	93 (86; 101)	1.81 × 10^−2^	96 (89; 105)	94 (88; 102)	0.14
insulin, µU/L ^b^	10.9 (7.1; 20.5)	8.5 (6.07; 14.2)	5.38 × 10^−4^	10.3 (6.5; 20.4)	8.8 (6.4; 15.8)	0.12
HOMA-IR ^b^	2.67 (1.53; 4.84)	1.94 (1.35; 3.31)	7.05 × 10^−4^	2.51 (1.51; 5.03)	2.07 (1.44; 4.09)	0.07
total cholesterol, mmol/L ^b^	4.74 (4.19; 5.26)	4.57 (4.44; 5.07)	2.67 × 10^−2^	4.67 (4.55; 5.14)	4.43 ± 0.77	0.06
triglycerides, mmol/L ^b^	114 (78; 154)	98 (68; 131)	4.59 × 10^−3^	101 (76; 140.5)	98 (67; 151)	0.26
inflammatory markers						
CRP, ng/mL ^b^	3.45 (1.7; 7.38)	2.9 (1.5; 6.2)	0.14	2.7 (1.5; 6.1)	3.5 (1.5; 7.45)	0.37
IL-6, pg/mL ^b^	4.1 (2.9; 5.9)	3.5 (2.6; 5.1)	2.4 × 10^−2^	3.7 (2.65; 4.9)	3.5 (2.6; 5.3)	0.79
metabolic comorbidities						
obesity, *n* (%) ^a^	109 (53.96)	71 (35.15)	2.13 × 10^−4^	69 (37.7)	55 (30.05)	0.15
type 2 diabetes, *n* (%) ^a^	33 (16.75)	20 (10.00)	0.07	23 (12.64)	18 (9.84)	0.5
hyperlipidaemia, *n* (%) ^a^	69 (35.38)	46 (23.35)	1.22 × 10^−2^	69 (35.38)	41 (22.53)	0.06
arterial hypertension, *n* (%) ^a^	91 (47.52)	71 (35.68)	2.12 × 10^−2^	68 (37.57)	58 (31.69)	0.29

Data was tested for normal distribution using Shapiro–Wilk test. Normally distributed data are presented as mean ± standard deviation, skewed data as mean (interquartile range), and categorical variables as counts (percentage). *p*: ^a^ Univariate statistical significance was evaluated using χ^2^ Test. ^b^ Univariate statistical significance was evaluated using Mann–Whitney U Test. Because of missing values, number of subjects can differ. Variable highlighted in bold will be used as a confounder in all the following analyses. Abbreviations: HOMA-IR = homeostatic model assessment for insulin resistance; CRP = *C*-reactive protein; IL-6 = interleukin 6.

**Table 2:** Impact of metabolic alterations on allergy occurrence.

	Contact Allergy		Hay Fever	
	OR (LCI; UCI)	*p*	OR (LCI; UCI)	*p*
metabolic markers				
glucose, mg/dL	1.00 (0.99; 1.00)	0.83	1.00 (0.99; 1.01)	0.46
insulin, µU/L	1.00 (0.99; 1.00)	0.39	1.01 (0.99; 1.03)	0.22
HOMA-IR	1.00 (0.99; 1.03)	0.48	1.03 (0.98; 1.09)	0.21
total cholesterol, mmol/L	1.33 (0.98; 1.82)	0.07	1.49 (1.04; 2.14)	3.05 × 10^−2^
triglycerides, mmol/L	1.00 (0.99; 1.00)	0.06	1.00 (1.00; 1.00)	0.80
inflammatory markers				
CRP, ng/mL	1.00 (0.96; 1.04)	0.93	0.94 (0.88; 1.00)	0.05
IL-6, pg/mL	1.04 (0.99; 1.09)	0.17	0.95 (0.89; 1.00)	0.05
metabolic comorbidities				
+ type 2 diabetes, *n* (%)	1.32 (0.7; 2.48)	0.39	1.18 (0.59; 2.34)	0.64
+ hyperlipidaemia, *n* (%)	1.62 (1.03; 2.55)	3.57 × 10^−2^	1.63 (1.01; 2.61)	4.15 × 10^−2^
+ arterial hypertension, *n* (%)	1.24 (0.79; 1.24)	0.35	1.18 (0.74; 1.9)	0.49

*p*: Statistical significance was evaluated using BMI-adjusted binomial logistic regression. For the assessment of metabolic comorbidities, absence and presence (+) of disease have been compared. *Abbreviations*: OR = odds ratio; LCI = lower confidence interval (5%); UCI = upper confidence interval (95%); HOMA-IR = homeostatic model assessment for insulin resistance; CRP = *C*-reactive protein; IL-6 = interleukin 6.

**Table 3:** Dietary characterization of the study population.

	Contact Allergy			Hay Fever		
	Cases	Controls	*p*	Cases	Controls	*p*
subjects, *n* (%) ^a^	202 (50)	202 (50)	1.0	183 (50)	183 (50)	1.0
taste perception						
saline, 0–300 ^b^	123 (105; 165)	123 (104.5; 171.25)	0.74	123 (99.25; 169)	123 (101.5; 165.25)	0.63
bitter, 0–300 ^b^	82 (42; 123)	81.75 (41; 122)	0.16	81.5 (42; 114)	67 (41; 102)	0.12
overall intake						
energy, kJ/day ^b^	8578 (7175; 10,330)	8164 (6821; 9552)	0.05	9076 (7227; 11,199)	9197 (7356; 11,194)	0.83
food, g/day	3607 (2979; 4394)	3395 (2776; 3995)	2.39 × 10^−3^	3609 (2802; 4416)	3718 (2989; 4395)	0.57
macronutrient intake						
carbohydrates, EN% ^b^	42.64 (39.31; 47.21)	41.43 (38.75; 45.36)	2.91 × 10^−2^	41.32 ± 6.96	41.16 (37.97; 45.28)	0.68
protein, EN% ^b^	14.84 ± 2.01	14.52 (13.23; 15.9)	0.24	14.63 (13.02; 15.98)	14.49 ± 2.15	0.58
fats, EN% ^b^	38.32 (34.91; 41.44)	39.68 (36.31; 43.07)	1.41 × 10^−2^	38.88 (36.47; 42.92)	40.16 (36.47; 42.98)	0.55
fibre, g/day ^b^	21.7 (18.94; 25.82)	22.15 (19.44; 25.84)	0.38	21.68 ± 5.36	21.29 (18.72; 24.63)	0.88
micronutrient intake						
vitamins, g/day ^b^	7.78 (6.36; 9.59)	7.48 (6.46; 9.07)	0.32	6.62 (5.05; 8.89)	7.26 (5.23; 9.25)	0.32
minerals, g/day ^b^	16.83 (15.5; 17.93)	16.6 (15.68; 17.54)	0.75	16.58 (15.53; 17.91)	16.7 (15.67; 17.91)	0.77

Data was tested for normal distribution using Shapiro–Wilk test. Normally distributed data are presented as mean ± standard deviation, skewed data as man (interquartile range), and categorical variables as counts (percentage). *p*: ^a^ Univariate statistical significance was evaluated using χ^2^ Test. ^b^ Univariate statistical significance was evaluated using Mann–Whitney U Test.

**Table 4:** Physical activity, sleep, and smoking habit characterization of the study population.

	Contact Allergy			Hay Fever		
	Cases	Controls	*p*	Cases	Controls	*p*
subjects, *n* (%) ^a^	202 (50)	202 (50)	1.0	183 (50)	183 (50)	1.0
physical activity						
sports, min/week ^b^	172.5 (39.38; 330)	210 (60; 390)	0.16	210 (90; 360)	300 (120; 465)	4.84 × 10^−3^
everyday activity, min/week ^b^	975 (588.75; 1620)	1039 (634; 1620)	0.51	907.5 (480; 1305)	990 (525; 1552.5)	0.14
watch TV, h/week ^b^	21 (14; 28)	14 (10.5; 21.0)	4.16 × 10^−2^	14 (10.5; 21)	21 (14; 28)	0.7
duration of sleep						
total sleep, h/24 h ^b^	7.25 (7; 8)	7 (7; 8)	0.42	7 (7;8)	7.5 (7; 8.12)	2.13 × 10^−3^
night sleep, h ^b^	7 (6; 8)	7 (6; 8)	0.54	7 (6; 8)	7 (7; 8)	8.13 × 10^−3^
day sleep, h ^b^	0 (0; 1)	0 (0; 0.5)	1.89 × 10^−2^	0 (0; 0.5)	0 (0; 0.5)	0.53
smoking habit						
+ smoker, *n* (%) ^a^	34 (17.71)	41 (21.13)	0.47	32 (17.78)	29 (16.11)	0.78

Data was tested for normal distribution using Shapiro–Wilk test. Normally distributed data are presented as mean ± standard deviation, skewed data as mean (interquartile range), and categorical variables as counts (percentage). *p*: ^a^ Univariate statistical significance was evaluated using χ^2^ Test. ^b^ Univariate statistical significance was evaluated using Mann–Whitney U Test.

**Table 5:** Metabolic pathways associated to contact allergy and hay fever.

Contact Allergy				Hay Fever			
Pathway	Metabolites	Impact	*p*	Pathway	Metabolites	Impact	*p*
Glycerophospholipid metabolism	- Phosphatidylethanolamine - Phosphatidylcholine - 1-Acyl-sn-glycero-3-phosphocholine	0.22	2.19 × 10^−3^	Glycerophospholipid metabolism	- Phosphatidylethanolamine - Phosphatidylcholine	0.2	0.06
α-Linolenic acid metabolism	- OPC6-CoA - Phosphatidylcholine	0.33	4.09 × 10^−3^	α-Linolenic acid metabolism	- Phosphatidylcholine	0	0.13
Phenylalanine, tyrosine and tryptophan biosynthesis	- L-Phenylalanine	0.5	3.06 × 10^−2^	Phenylalanine, tyrosine and tryptophan biosynthesis	- L-Phenylalanine	0.5	4.06 × 10^−2^

Metabolic pathways were identified using MetaboAnalyst 5.0. Enrichment method was set at hypergeometric test (*p* values) and topology analysis at relative-betweenness centrality (impact). Analysis was carried out within the KEGG pathway library.

## Data Availability

The datasets generated and analysed during the current study are available from the corresponding author on reasonable request.

## References

[1] Langen U., Schmitz R., Steppuhn H. (2013). Häufigkeit allergischer Erkrankungen in Deutschland: Ergebnisse der Studie zur Gesundheit Erwachsener in Deutschland (DEGS1). Bundesgesundheitsblatt Gesundh. Gesundh..

[2] World Health Organization Obesity and Overweight. https://www.who.int/news-room/fact-sheets/detail/obesity-and-overweight.

[3] Sybilski A.J., Raciborski F., Lipiec A., Tomaszewska A., Lusawa A., Furmańczyk K., Krzych-Fałta E., Komorowski J., Samoliński B. (2015). Obesity—A risk factor for asthma, but not for atopic dermatitis, allergic rhinitis and sensitization. Public Health Nutr..

[4] Visness C.M., London S.J., Daniels J.L., Kaufman J.S., Yeatts K.B., Siega-Riz A.-M., Liu A.H., Calatroni A., Zeldin D.C. (2009). Association of obesity with IgE levels and allergy symptoms in children and adolescents: Results from the National Health and Nutrition Examination Survey 2005–2006. J. Allergy Clin. Immunol..

[5] Abbassi S., Capitle E., Trivedi R., Wolff A.H. (2021). Obesity is associated with an increased prevalence of penicillin allergy. Ann. Allergy Asthma Immunol..

[6] Dias de Castro E., Pinhão S., Paredes S., Cernadas J.R., Ribeiro L. (2021). Obesity markers in patients with drug allergy and body fat as a predictor. Ann. Allergy Asthma Immunol..

[7] Boeing H., Wahrendorf J., Becker N. (1999). EPIC-Germany—A source for studies into diet and risk of chronic diseases. European Investigation into Cancer and Nutrition. Ann. Nutr. Metab..

[8] Nöthlings U., Hoffmann K., Bergmann M.M., Boeing H. (2007). Fitting portion sizes in a self-administered food frequency questionnaire. J. Nutr..

[9] Noethlings U., Hoffmann K., Bergmann M.M., Boeing H. (2003). Portion size adds limited information on variance in food intake of participants in the EPIC-Potsdam study. J. Nutr..

[10] Rühlemann M.C., Hermes B.M., Bang C., Doms S., Moitinho-Silva L., Thingholm L.B., Frost F., Degenhardt F., Wittig M., Kässens J. (2021). Genome-wide association study in 8956 German individuals identifies influence of ABO histo-blood groups on gut microbiome. Nat. Genet..

[11] Henneke L., Schlicht K., Andreani N.A., Hollstein T., Demetrowitsch T., Knappe C., Hartmann K., Jensen-Kroll J., Rohmann N., Pohlschneider D. (2022). A dietary carbohydrate—Gut Parasutterella—Human fatty acid biosynthesis metabolic axis in obesity and type 2 diabetes. Gut Microbes.

[12] Matyash V., Liebisch G., Kurzchalia T.V., Shevchenko A., Schwudke D. (2008). Lipid extraction by methyl-tert-butyl ether for high-throughput lipidomics. J. Lipid Res..

[13] Kind T., Fiehn O. (2007). Seven Golden Rules for heuristic filtering of molecular formulas obtained by accurate mass spectrometry. BMC Bioinform..

[14] Heinsen F.-A., Fangmann D., Müller N., Schulte D.M., Rühlemann M.C., Türk K., Settgast U., Lieb W., Baines J.F., Schreiber S. (2016). Beneficial Effects of a Dietary Weight Loss Intervention on Human Gut Microbiome Diversity and Metabolism Are Not Sustained during Weight Maintenance. Obes. Facts.

[15] Ho D.E., Imai K., King G., Stuart E.A. (2011). MatchIt: Nonparametric Preprocessing for Parametric Causal Inference. J. Stat. Soft..

[16] Lusi E.A., Di Ciommo V.M., Patrissi T., Guarascio P. (2015). High prevalence of nickel allergy in an overweight female population: A pilot observational analysis. PLoS ONE.

[17] Watanabe M., Masieri S., Costantini D., Tozzi R., de Giorgi F., Gangitano E., Tuccinardi D., Poggiogalle E., Mariani S., Basciani S. (2018). Overweight and obese patients with nickel allergy have a worse metabolic profile compared to weight matched non-allergic individuals. PLoS ONE.

[18] Manti S., Cuppari C., Marseglia L., D’Angelo G., Arrigo T., Gitto E., Leonardi S., Salpietro C. (2017). Association between Allergies and Hypercholesterolemia: A Systematic Review. Int. Arch. Allergy Immunol..

[19] Kim S.Y., Sim S., Park B., Kim J.-H., Choi H.G. (2016). High-Fat and Low-Carbohydrate Diets Are Associated with Allergic Rhinitis But Not Asthma or Atopic Dermatitis in Children. PLoS ONE.

[20] Trak-Fellermeier M.A., Brasche S., Winkler G., Koletzko B., Heinrich J. (2004). Food and fatty acid intake and atopic disease in adults. Eur. Respir. J..

[21] Sawane K., Nagatake T., Hosomi K., Hirata S.-I., Adachi J., Abe Y., Isoyama J., Suzuki H., Matsunaga A., Fukumitsu S. (2019). Dietary Omega-3 Fatty Acid Dampens Allergic Rhinitis via Eosinophilic Production of the Anti-Allergic Lipid Mediator 15-Hydroxyeicosapentaenoic Acid in Mice. Nutrients.

[22] Kunisawa J., Arita M., Hayasaka T., Harada T., Iwamoto R., Nagasawa R., Shikata S., Nagatake T., Suzuki H., Hashimoto E. (2015). Dietary ω3 fatty acid exerts anti-allergic effect through the conversion to 17,18-epoxyeicosatetraenoic acid in the gut. Sci. Rep..

[23] Roxbury C.R., Qiu M., Shargorodsky J., Lin S.Y. (2018). Association between allergic rhinitis and poor sleep parameters in U.S. adults. Int. Forum Allergy Rhinol..

[24] Silva D., Moreira A. (2015). The role of sports and exercise in allergic disease: Drawbacks and benefits. Expert Rev. Clin. Immunol..

[25] Helenius I., Haahtela T. (2000). Allergy and asthma in elite summer sport athletes. J. Allergy Clin. Immunol..

[26] Muscella A., Stefàno E., Marsigliante S. (2020). The effects of exercise training on lipid metabolism and coronary heart disease. Am. J. Physiol. Heart Circ. Physiol..

[27] Poggiogalle E., Jamshed H., Peterson C.M. (2018). Circadian regulation of glucose, lipid, and energy metabolism in humans. Metabolism.

[28] Pascal M., Perez-Gordo M., Caballero T., Escribese M.M., Lopez Longo M.N., Luengo O., Manso L., Matheu V., Seoane E., Zamorano M. (2018). Microbiome and Allergic Diseases. Front. Immunol..

[29] Belkaid Y., Hand T.W. (2014). Role of the microbiota in immunity and inflammation. Cell.

[30] Watts A.M., West N.P., Zhang P., Smith P.K., Cripps A.W., Cox A.J. (2021). The Gut Microbiome of Adults with Allergic Rhinitis Is Characterised by Reduced Diversity and an Altered Abundance of Key Microbial Taxa Compared to Controls. Int. Arch. Allergy Immunol..

[31] Bäckhed F., Fraser C.M., Ringel Y., Sanders M.E., Sartor R.B., Sherman P.M., Versalovic J., Young V., Finlay B.B. (2012). Defining a healthy human gut microbiome: Current concepts, future directions, and clinical applications. Cell Host Microbe.

[32] Ohnmacht C. (2016). Microbiota, regulatory T cell subsets, and allergic disorders. Allergo J. Int..

[33] Bona M.D., Torres C.H.d.M., Lima S.C.V.C., Lima A.A.M., Maciel B.L.L. (2021). Intestinal barrier function in obesity with or without metabolic syndrome: A systematic review protocol. BMJ Open.

[34] Majamaa H., Isolauri E. (1996). Evaluation of the gut mucosal barrier: Evidence for increased antigen transfer in children with atopic eczema. J. Allergy Clin. Immunol..

[35] Ukabam S.O., Mann R.J., Cooper B.T. (1984). Small intestinal permeability to sugars in patients with atopic eczema. Br. J. Dermatol..

